# Outcomes of Combined Hepatectomy and Intraoperative Ablation for Multiple Colorectal Liver Metastases: A Single-Center Retrospective Study

**DOI:** 10.3390/cancers18203386

**Published:** 2026-10-20

**Authors:** Tsukasa Aihara, Shinichi Ikuta, Takayoshi Nakajima, Shinta Nagano, Toshimitsu Iwasaki, Hidenori Yanagi, Naoki Yamanaka

**Affiliations:** Meiwa Hospital, Nishinomiya 663-8186, Japan; ikuta@meiwa-hospital.com (S.I.); nakajima.t@meiwa-hospital.com (T.N.); nagano.s@meiwa-hospital.com (S.N.); iwasaki.t@meiwa-hospital.com (T.I.); yanagih@meiwa-hospital.com (H.Y.); nyamana@meiwa-hospital.com (N.Y.)

**Keywords:** colorectal liver metastases, hepatectomy, post-hepatectomy liver failure, limited liver reserve, hybrid hepatectomy, parenchymal-sparing strategy

## Abstract

**Introduction**: Radical hepatectomy is the standard curative treatment for colorectal liver metastases (CRLMs). However, extensive resection may be contraindicated in patients with insufficient liver reserve or anatomically complex disease. Although ablation therapy is generally not recommended as a primary curative modality in current guidelines, intraoperative ablation may facilitate parenchymal-sparing, curative-intent treatment in selected high-risk patients. **Methods**: This retrospective cohort study included 118 patients who underwent curative-intent hepatectomy for multiple CRLM. Patients were categorized into hepatectomy-only (Hx) and hepatectomy plus intraoperative ablation (Hx + Abl) groups, and propensity score matching (PSM) generated 29 balanced pairs. Overall survival (OS) and hepatic progression-free survival (HPFS) were compared using a log-rank test. Cox regression identified predictors of OS, and receiver operating characteristic (ROC) analysis evaluated tumor size associated with local recurrence after ablation. **Results**: Before PSM, the HX + Abl group showed higher tumor burden and Beppu scores, and demonstrated inferior OS and HPFS. After PSM, oncological outcomes were comparable. Perioperative outcomes, including major complications and post-hepatectomy liver failure (PHLF), were similar. Preoperative chemotherapy was an independent predictor of OS. Among 99 ablated tumors, local recurrence occurred in 12 lesions, and ROC analysis identified tumor size ≤ 15mm as the optimal cutoff predicting local recurrence. **Conclusions**: In selected high-risk CRLM patients, hepatectomy with intraoperative ablation had no statistically significant difference in OS and HPFS compared with hepatectomy alone without increasing major complications or PHLF.

## Simple Summary

This study evaluated whether adding intraoperative ablation to hepatectomy can provide safe and effective treatment for patients with multiple colorectal liver metastases (CRLMs). A total of 118 patients underwent curative-intent hepatectomy, and 29 matched pairs were created using propensity score matching. Patients receiving hepatectomy plus ablation had no statistically significant difference in overall survival and hepatic progression-free survival compared with hepatectomy alone after matching. Perioperative outcomes, including major complications and post-hepatectomy liver failure, were also comparable. Among 99 ablated tumors, local recurrence occurred mainly in lesions larger than 15 mm. These findings suggest that combining resection for superficial tumors with ablation for deep lesions can preserve liver tissue while maintaining oncologic effectiveness. This hybrid, parenchymal-sparing strategy may be a useful option for high-risk patients with limited liver reserve or anatomically complex disease, especially when targeting small tumors.

## 1. Introduction

Colorectal liver metastases (CRLMs) remain a major determinant of prognosis in colorectal cancer. Complete resection (R0 hepatectomy) is the foundation of curative-intent treatment. Prognostic scoring systems, such as the Beppu [1], the ras-Beppu [2], and the GAME scores [3], have facilitated refined stratification of long-term outcomes after hepatectomy. These models incorporate tumor burden, tumor biology, including RAS mutation status [2,3], and liver function reserve, and are widely used to identify patients at high risk of recurrence or post-hepatectomy liver failure (PHLF) [1,4]. Patients classified as high-risk by these scores often present with multiple bilobar metastases, aggressive tumor biology, or impaired liver function, and consequently have inferior overall survival (OS) and increased perioperative risk. Current international guidelines consistently position surgical resection as the preferred curative-intent treatment for resectable CRLM. Across the ESMO, NCCN, and JSCCR guidelines [5,6,7], complete resection of all visible disease is regarded as offering the best chance of long-term survival, whereas thermal ablation is considered a selective adjunct rather than a primary curative modality. All three guidelines acknowledge that thermal ablation is an appropriate local therapy for small lesions (≤3 cm) or anatomically challenging metastases where parenchymal preservation is desirable, and that combined resection and ablation may be used in selected patients with multifocal or deep lesions when complete local control can be achieved without compromising postoperative liver function.

These evolving recommendations have been reinforced by recent high-quality evidence. The COLLISION randomized controlled trial demonstrated the non-inferiority of thermal ablation compared with surgical resection for small (≤3 cm) CRLM, validating ablation as a curative-intent option in carefully selected patients [8]. As a result, ablation-assisted parenchymal-sparing strategies have gained increasing acceptance as viable options for patients who would otherwise require extensive hepatectomy or be at risk of inadequate liver reserve.

In parallel with these guideline-aligned developments, the role of parenchymal-sparing strategies combining hepatectomy with intraoperative ablation has gained increasing traction. Recent studies have demonstrated that combining limited hepatectomy with microwave ablation (MWA) or radiofrequency ablation (RFA) can achieve long-term outcomes comparable to those of hepatectomy alone in selected patients with multiple CRLMs [9,10,11,12]. Thus, ablation-assisted surgery may expand curative-intent options for patients who would otherwise require extensive hepatectomy or be deemed unsuitable for resection. However, most previous studies included heterogeneous patient populations and did not specifically evaluate outcomes in patients with high Beppu, ras-Beppu, or GAME scores. Consequently, the benefit of ablation-assisted hepatectomy in these high-risk groups remains unclear. To address these gaps, we aimed to evaluate perioperative and oncological outcomes of hepatectomy combined with intraoperative ablation compared with hepatectomy alone in patients with multiple CRLMs. Importantly, our cohort included a substantial proportion of high-risk patients defined by Beppu, ras-Beppu, and GAME scores, allowing a focused assessment of ablation-assisted hepatectomy in this clinically challenging population. Using propensity score matching (PSM), we sought to evaluate whether this combined strategy provides acceptable oncological outcomes without increasing postoperative morbidity, thereby offering a viable option for patients with limited liver reserve, anatomically complex disease, or high prognostic risk scores.

## 2. Methods

### 2.1. Patient Selection

This retrospective cohort study was conducted at Meiwa Hospital. Patients who underwent curative-intent hepatectomy or combined hepatectomy and ablation for ≥2 CRLMs between January 2014 and December 2022 were included in analysis. The exclusion criteria were as follows: (1) recurrent CRLM; (2) controlled extrahepatic disease according to RECIST criteria; and (3) no curative surgery. Extrahepatic disease was included when controlled and deemed resectable or treatable. Patients were categorized into two groups: hepatectomy alone and hepatectomy plus intraoperative ablation.

Resectability was defined from Yamanaka’s prediction score, which is a composite model developed from postoperative outcomes of patients undergoing hepatectomy [13,14]. The score estimates the safety extent of liver resection by integrating the planned liver resection rate (PHRR), ICG clearance (ICG R15), and patient age (Age) to estimate the safety extent of hepatectomy. The score is calculated using the following regression equation:

“Prediction score (PS)” = −84.6 + 0.933 × “PHRR” + 1.11 × “ICG R15” + 0.999 × “Age”

Based on the original validation study, a PS ≤ 45 is classified as the safe zone, indicating that major hepatectomy can be performed with acceptable risk of post-hepatectomy liver failure. Herein, the strategy of ablation combined with liver resection was standardized as follows. First, tumors located on the liver surface were treated by wedge resection. Second, for deeply situated tumors, anatomical segmentectomy was performed when multiple lesions were present within the same segment. Third, when a single deep lesion was confined to one segment, ablation was selected when the Yamanaka score exceeded the safe threshold (PS > 45), indicating that anatomical resection would surpass the safe resection limit and jeopardize postoperative liver function. Microwave ablation (MWA) or radiofrequency ablation (RFA) was performed under intraoperative ultrasound guidance. Technical success and ablative margins were evaluated using contrast-enhanced intraoperative ultrasound and postoperative CT/MRI. Device type, power settings, ablation time, and antenna positions were recorded. A minimum ablative margin of ≥5 mm was secured. MWA was performed using the Microtaze system, whereas RFA (Cool Tip) was selected when the tumor was located within 5 mm of major vessels.

### 2.2. Variables

Baseline characteristics included age, sex, albumin, total bilirubin, aspartate aminotransferase (AST), platelet count, prothrombin time, indocyanine green retention at 15 min (ICG-R15), tumor markers (CEA, CA19-9), RAS mutation status, primary tumor T/N stage, tumor number and size, timing of metastasis, extrahepatic disease, and peri- and postoperative chemotherapy. As indicators of hepatic functional reserve, the ALBI score and APRI score were calculated. Measures of tumor aggressiveness in CRLM, including the Beppu, ras-Beppu, and GAME scores, were also determined. The Beppu score was calculated as the sum of six preoperative factors: synchronous metastasis, positive primary lymph nodes, tumor number, maximum tumor diameter, extrahepatic disease at liver resection, and serum CA19 9 ≥ 100 U/mL. The ras-Beppu score further integrates RAS mutation status into the Beppu classification to refine recurrence risk stratification. The GAME score combines tumor size, tumor number, extrahepatic disease, CEA level, and RAS mutation status to generate a composite genetic–morphologic risk score.

Perioperative variables included operation time, blood loss, resected liver weight and ratio, major complications (Clavien–Dindo ≥ III) [15], and PHLF (ISGLS criteria) [16]. Post-hepatectomy liver failure (PHLF) was assessed according to the International Study Group of Liver Surgery (ISGLS) criteria, which define PHLF as an increase or insufficient improvement in serum bilirubin levels together with prolonged prothrombin time (INR) on or after postoperative day 5. The severity is graded from A to C based on the clinical impact and the need for supportive interventions.

Systemic chemotherapy regimens were classified according to the backbone cytotoxic agent. Oxaliplatin-based therapy (*n* = 52) included FOLFOX, SOX, XELOX, and FOLFOXIRI. Irinotecan-based therapy (*n* = 22) included FOLFIRI, IRIS, and XELIRI. S1-based therapy (*n* = 8) included S1 or UFT, whereas 5 fluorouracil-based therapy (*n* = 1) consisted of 5 FU monotherapy. Capecitabine-based therapy (*n* = 4) included XELOX and XELIRI. Trifluridine/tipiracil-based therapy (*n* = 1) was classified as the TAS 102 group. Biologic agents (bevacizumab, panitumumab, cetuximab, and ramucirumab) were recorded separately but did not affect backbone classification.

### 2.3. Outcomes

Primary endpoints were OS and hepatic progression-free survival (HPFS). HPFS was defined as the time to any intrahepatic recurrence, including recurrence at the ablation zone, or death. Extrahepatic recurrence alone was not considered an HPFS event. Local recurrence after ablation was defined as local tumor progression, characterized by nodular or crescent enhancement at the ablation margin on contrast-enhanced CT or MRI.

Follow up imaging consisted of contrast-enhanced CT or MRI every 3–4 months for 2 years and every 6 months thereafter.

OS and HPFS were estimated using the Kaplan–Meier method and compared using the log-rank test. Receiver operating characteristic (ROC) analysis was performed to determine the optimal tumor size cutoff for predicting local recurrence after ablation. Subgroup analyses were performed according to Beppu, ras-Beppu, and GAME scores, which were dichotomized into high and low risk categories based on previously reported cutoffs or median values. Cox regression models including interaction terms between treatment modality (Hx vs. Hx + Abl) and each risk category were used to explore heterogeneity of treatment effect.

### 2.4. Statistical Analysis

Continuous variables were expressed as mean ± standard deviation and compared between groups using Student’s *t*-test or the Mann–Whitney U-test. Categorical variables were analyzed using the chi-square test or Fisher’s exact test, as appropriate. *p*-values < 0.05 were considered significant. To reduce selection bias, a 1:1 PSM was performed using clinically relevant covariates. This yielded 29 matched pairs (HX *n*—29; Hx + Abl *n*—29), with balanced baseline characteristics including age, RAS mutation status, primary tumor N stage ≥ 1, tumor number and size, timing of metastasis, extrahepatic disease, and tumor markers (CEA and CA19-9). Matching was performed using 1:1 nearest neighbor matching without replacement, with a caliper of 0.20 SD of the logit of the propensity score. Covariate balance was assessed using standardized mean differences (SMDs), with <0.10 indicating adequate balance.

To further adjust for residual confounding, inverse probability of treatment weighting (IPTW) analysis was performed using the same covariates as the propensity score model. Weighted Cox proportional hazards regression with robust variance estimation was used to evaluate treatment effects on overall survival (OS) and hepatic progression-free survival (HPFS). Covariate balance after weighting was confirmed by SMD < 0.10. OS and hepatic HPFS were estimated using the Kaplan–Meier test, and differences between groups were evaluated using the log-rank test. Univariate analyses for OS were conducted using the Cox proportional hazards model. Preoperative variables with *p* < 0.10 in univariate analysis were entered into the multivariate Cox regression model to identify independent prognostic factors. Postoperative variables (PHLF, postoperative chemotherapy, intrahepatic recurrence) were excluded to avoid post-treatment and immortal time bias. For ablated lesions, diagnostic performance of tumor size for predicting local recurrence was evaluated using an ROC curve. The optimal cutoff was determined using the Yoden index, and AUC values were reported with 95% CIs. Lesion-level analyses for local recurrence were conducted exclusively in the ablation cohort, as local recurrence is a lesion-specific outcome relevant only to ablated lesions. Frailty and generalized estimating equation (GEE) models were applied to account for potential intra-patient correlation among multiple ablated lesions. The hepatectomy cohort was excluded from these models because local recurrence could not be defined at the lesion-level. All statistical analyses were performed using EZR (Easy R version 1.70; Saitama Medical Center, Jichi Medical University, Japan) and RStudio (R version 4.6.1; R Foundation for Statistical Computing, Vienna, Austria).

## 3. Results

A total of 131 patients underwent hepatectomy during the study period. Among them, 13 patients were excluded due to repeat hepatectomy (7), progression of extrahepatic lesion (4), R2 resection (2). The remaining 118 patients included 83 hepatectomy alone and 35 hepatectomy plus intraoperative ablation patients. The patient selection flow is illustrated in Figure 1. Tumor response to preoperative chemotherapy was assessed using RECIST version 1.1. Partial response (PR), stable disease (SD), and progressive disease (PD) were observed in 38, 29, and 33 regimens, respectively. PR was mainly achieved with oxaliplatin-based or irinotecan-based combinations with biologics, whereas SD was frequently associated with TAS 102 or oxaliplatin-based therapy. PD occurred predominantly in SOX based and irinotecan-based regimens. Treatment exposure ranged from 2 to 12 cycles.

### 3.1. Patient Characteristics

Before matching, the Hx + Abl group had a significantly higher tumor number (8.4 vs. 4.5, *p* < 0.001) and Beppu score (13.5 vs. 11.0, *p* = 0.003), and tumor burden score (11.5 vs. 8.1, *p* = 0.006) indicating more aggressive oncological characteristics. AST was also higher (30.1 vs. 24.6, *p* = 0.019), although ALBI score and other liver function parameters were comparable. After PSM, all major baseline variables including tumor number (6.5 vs. 6.1, *p* = 0.779), Beppu score (12.9 vs. 12.8, *p* = 0.925), tumor burden score (9.7 vs. 9.3, *p* = 0.785), and GAME score (3.3 vs. 3.2, *p* = 0.918) were well balanced. AST remained significantly higher in the Hx + Abl group (29.9 vs. 21.5, *p* = 0.005), resulting in a higher APRI score (1.8 vs. 1.4, *p* = 0.024), whereas ALBI score showed no significant difference. The anatomical distribution of colorectal liver metastases showed that H1, H2, and H3 disease accounted for 48.6% (52/107), 42.1% (45/107), and 9.3% (10/107), respectively. After matching, the H classification remained balanced between groups, indicating comparable surgical extent and treatment strategy. Six Hx + Abl patients were excluded during propensity score matching due to a combination of higher tumor burden, elevated serum tumor markers, and younger age compared with the matched population. Overall, the matched cohort (*n* = 29 each) demonstrated balanced distributions across all the other evaluated variables, supporting the validity of the adjusted comparison of surgical oncological outcomes. Detailed baseline characteristics are summarized in Table 1.

### 3.2. Perioperative Outcomes

After matching, perioperative outcomes were comparable between groups. Operation time, blood loss, hospital stay, major complications, and PHLF incidence did not differ significantly. In addition, liver functional reserve indicators, including Yamanaka’s prediction and ALBI scores, were similar between the two groups after matching, indicating that hepatic functional status was balanced. Before matching, the future liver remnant (FLR) ratio tended to be higher in the HX + Abl group compared than in the Hx group (78.1% vs. 72.1%, *p* = 0.070). After PSM, the FLR ratio became significantly higher in the HX + Abl group, indicating that patients undergoing combined resection and ablation ultimately preserved a greater proportion of functional liver parenchyma. Perioperative outcomes and FLR data are presented in Table 1.

### 3.3. Survival Outcomes

OS and HPFS were inferior in the Hx + Abl group before PSM (OS median 27.9 vs. 38.4 months, *p* = 0.097; HPFS median 7.0 vs. 11.0 months, *p* = 0.027). The 3-year OS rates were 55.4% in the Hx group and 43.4% in the Hx + Abl group. After PSM, survival outcomes were comparable, with no significant differences in OS (median 44.5 vs. 36.8 months, *p* = 0.907) or HPFS (median 7.0 vs. 8.0 months, *p* = 0.581). The 3-year OS rates after matching were 56.0% in the Hx group and 51.9% in the Hx + Abl group. Kaplan–Meier curves for OS and HPFS before and after matching are shown in Figure 2. To further adjust for residual confounding, inverse probability of treatment weighting (IPTW) was applied using the same covariates as the propensity score model. After weighting, covariate balance improved markedly, with most standardized mean differences below 0.1. Weighted Cox regression showed no significant difference in overall survival between the Abl and Hx groups (HR = 1.28, 95% CI 0.65–2.52, *p* = 0.48; concordance = 0.55) or in hepatic progression-free survival (HR = 1.24, 95% CI 0.72–2.16, *p* = 0.44; concordance = 0.51). These results indicate that, after adjustment for baseline characteristics, treatment modality was not independently associated with either overall survival or hepatic recurrence.

### 3.4. Risk Factor Analysis

Multivariate Cox analysis identified preoperative chemotherapy as an independent predictor of OS (HR 3.324, 95% CI 1.537–7.1888, *p* = 0.002). Treatment modality (Hx vs. Hx + Abl) was not associated with OS. The results of the multivariate analysis are summarized in Table 2.

### 3.5. Local Recurrence After Ablation

Among 99 ablated tumors, 12 (12.1%) developed local recurrence. Lesion-level frailty analysis identified tumor size as a significant predictor. ROC analysis yielded an AUC of 0.791 (95% CI: 0.665–0.916), with an exploratory cutoff of 15 mm. Local recurrence occurred in 7/18 lesions > 15 mm and 5/81 lesions ≤ 15 mm. The ROC curve is shown in Figure 3. Lesion-level frailty and GEE analyses were performed exclusively in the ablation cohort, since local recurrence is a lesion-specific event applicable only to ablated lesions. In the hepatectomy cohort, lesion-level recurrence could not be evaluated; therefore, these models were not applied. The ablation-specific analyses demonstrated that tumor size was not a statistically significant predictor of local recurrence, consistent with the ROC-based exploratory cutoff of 15 mm.

### 3.6. Subgroup Analyses

To evaluate whether treatment effects differed across prognostic strata, subgroup analyses were performed according to Beppu, ras-Beppu, and GAME scores. Within each scoring system, OS and HPFS curves showed no statistically significant differences between Hx and Hx + Abl in either high or low risk categories (Beppu: OS *p* = 0.30, HPFS *p* = 0.70; ras-Beppu: OS *p* = 0.50, HPFS *p* = 0.90; GAME: OS *p* = 0.90, HPFS *p* = 0.60).

Cox models incorporating interaction terms between the treatment modality and each risk category demonstrated no significant interactions (Beppu interaction HR 1.43, *p* = 0.66; ras-Beppu interaction HR 1.37, *p* = 0.69; GAME interaction HR 2.29, *p* = 0.25), indicating that the relative treatment effect of hybrid hepatectomy remained consistent across prognostic strata. These results suggest that ablation-assisted hepatectomy does not confer inferior survival in high-risk patients, although the limited sample size restricts definitive conclusions.

## 4. Discussion

In this retrospective cohort study, we evaluated the oncological validity of combining hepatectomy with intraoperative ablation for multiple CRLM in patients exhibiting high tumor burden and elevated prognostic scores such as the Beppu, ras-Beppu, and GAME scores [1,2,3]. Before matching, the Hx + Abl group demonstrated inferior OS and HPFS, largely reflecting their significantly worse baseline oncological profile, including higher tumor number and higher the Beppu and ras-Beppu scores, rather than the impact of the combined surgical strategy itself. These prognostic models are validated predictors of recurrence and long-term outcomes after hepatectomy, and their imbalance between groups underscores the more aggressive tumor biology present in patients selected for ablation-assisted surgery.

After PSM, which successfully balanced key prognostic variables including tumor number and Beppu score, OS and HPFS were comparable between the Hx and Hx + Abl groups. Although this study was not designed to establish equivalence or non-inferiority, the adjusted results suggest that hybrid hepatectomy can achieve oncologic outcomes similar to hepatectomy alone in appropriately selected patients. This finding aligns with contemporary treatment guidelines and emerging evidence. Both ESMO and NCCN recognize thermal ablation as an acceptable local therapy for small lesions (≤3 cm) or anatomically challenging metastases where parenchymal preservation is desirable [5,6,7], and recent reports support its role in achieving durable local control [8]. Given that a major rationale for ablation-assisted hepatectomy is its potential utility in patients with high prognostic risk, we conducted subgroup analyses stratified by Beppu, ras-Beppu, and GAME scores (Supplementary Figure S1). Across all three scoring systems, OS and HPFS did not differ significantly between Hx and Hx + Abl within either high or low risk categories, and interaction testing revealed no heterogeneity of treatment effect. These findings indicate that the relative performance of hybrid hepatectomy was broadly consistent across prognostic strata [4,9,10,11,12]. However, these analyses were exploratory and limited by small subgroup sizes, and therefore cannot establish a definitive benefit in high-risk patients. Nonetheless, the absence of a detrimental interaction supports the feasibility of integrating ablation into parenchymal-sparing strategies even in patients with elevated Beppu, ras-Beppu, or GAME scores, provided that careful lesion selection is maintained.

Perioperative outcomes were comparable between groups after matching, with no significant differences in operation time, blood loss, hospital stay, major complications, or the incidence of PHLF. The significantly higher FLR ratio observed in the Hx + Abl group after matching highlights the parenchymal-sparing advantage of combining limited hepatectomy with ablation. PHLF remains a critical determinant of preoperative mortality, and its prediction has evolved from resection-volume-based models to more refined functional reserve. Early prediction models by Yamanaka et al. [13,14] relied on mortality from PHLF, whereas later studies adopted the standardized ISGLS definition [16]. Schindl et al. [17] identified insufficient FLR as the strongest predictor of clinically significant PHLF, and Kambakamba et al. [18] proposed an APRI + ALBI-based model integrating biochemical and volumetric parameters. Beyond perioperative safety, FLR preservation confers oncologic advantages: repeat local therapy after parenchymal-sparing hepatectomy improves survival [19,20,21,22]. Thus, the increased FLR ratio in the Hx + Abl cohort may offer long-term benefits by maintaining hepatic reserve without compromising short-term outcomes.

In this study, we demonstrated that a hybrid strategy combining surgical resection for superficial CRLM with thermal ablation for deep parenchymal lesions enables effective local tumor control while preserving the functional liver remnant. Comparative studies [4,8,9,10,11] and a GRADE-assessed meta-analysis [23] showed that hepatectomy with intraoperative ablation yields survival outcomes comparable to hepatectomy alone. The MAVERRIC trial [24] and subsequent analyses [21] confirmed the non-inferiority of microwave ablation (MWA) to resection for small (<3 cm) or deep-seated lesions. Although MWA may provide slightly lower local control for subcapsular tumors, it offers reduced invasiveness and fewer complications. Recent reviews highlight the broader benefits of parenchymal preservation. Pandanaboyana et al. [25] and Alvarez et al. [26] showed that parenchymal-sparing surgery decreases morbidity and facilitates repeat therapy, while Milazzo et al. [27] and Jung et al. [28] demonstrated that non-anatomic resections achieve comparable oncologic outcomes to anatomic resections with better functional preservation. Collectively, these findings support a tailored combination of resection and ablation to balance oncologic radicality with hepatic reserve, offering durable outcomes in complex or high-risk CRLM cases.

Local recurrence after ablation remains a clinical concern. In our cohort, ROC analysis identified 15mm as the optimal tumor size cutoff for predicting local recurrence. This threshold is consistent with prior reports indicating reduced ablation efficacy for larger lesions [29]; however, the analysis was based on a limited number of events and lesion-level data without independent validation. Therefore, the predictive performance may be optimistic, and the proposed cutoff should be interpreted cautiously. Most ablated lesions in our cohort were small (mean 10.8 mm), likely contributing to the observed acceptable local control. These findings emphasize the importance of careful lesion selection when integrating ablation into curative-intent surgical management, particularly in high-risk patients.

Chemotherapy-induced tumor downsizing can convert borderline lesions [30] into candidates for resection or ablation, making tumor size and treatment response central to parenchymal-sparing strategies. Integrating tumor shrinkage with FLR assessment provides a practical framework for selecting interventions that minimize PHLF risk while preserving the feasibility of repeat local therapy. Within this framework, ablation-assisted hepatectomy represents a rational option for high-risk patients with limited liver reserve or anatomically complex disease, provided that small lesions (≤15 mm) are appropriately targeted. Moreover, inflammatory markers have been shown to predict survival after ablation, underscoring the prognostic interplay between tumor biology and local treatment efficacy [31].

This study has some limitations that warrant further consideration. First, its retrospective design introduces inherent selection bias, despite applying PSM to balance key prognostic variables. Second, the study was conducted at a single center, which may have limited generalizability because institutional expertise, ablation techniques, and perioperative management vary across centers. Third, although the overall cohort included 118 patients, the matched same size was relatively small, particularly for subgroup analyses such as local recurrence after ablation. Fourth, treatment allocation was influenced by intraoperative judgment and anatomical complexity, which may not be fully captured by available covariates. Finally, the follow-up duration may not be sufficient to assess very-long-term survival or late recurrence patterns. Future multicenter prospective studies incorporating prognostic models such as the Beppu, ras-Beppu, and GAME scores are merited to validate these findings and refine patient selection criteria.

Moreover, the optimal contribution of chemotherapy-induced tumor downsizing and rigorous FLR evaluation in optimizing curative-intent strategies warrants further investigation. Integrating these factors into prospective designs may help clarify the role of ablation-assisted parenchymal-sparing surgery within contemporary CRLM treatment algorithms and support more precise, biology-driven selection of candidates for combined resection–ablation approaches.

## 5. Conclusions

In selected high-risk CRLM patients, hepatectomy with intraoperative ablation achieved no statistically significant difference in OS and HPFS compared with hepatectomy alone after adjustment for baseline oncological factors without increasing major complications or PHLF. A hybrid strategy combining resection for superficial lesions and ablation for deep parenchymal lesions provides effective local tumor control while preserving liver function.

## Figures and Tables

**Figure 1:**
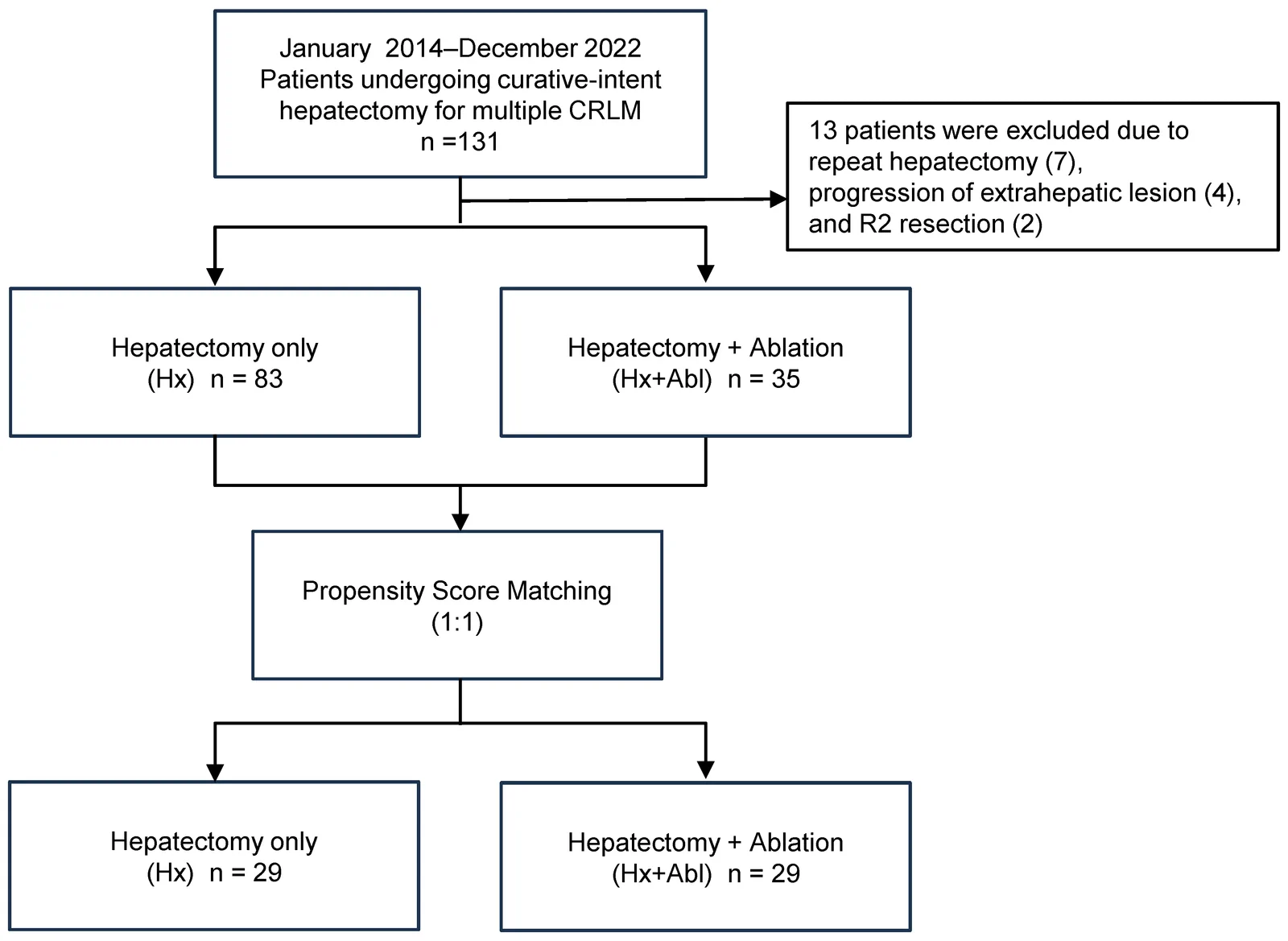
Inclusion criteria: Patients who underwent curative-intent hepatectomy for multiple colorectal liver metastases (CRLM) between January 2014 and December 2022 at a single center. Patients were divided into hepatectomy-only (Hx, *n* = 83) and hepatectomy plus ablation (Hx + Abl, *n* = 35) groups. Propensity score matching (1:1) was performed to yield 29 matched pairs.

**Figure 2:**
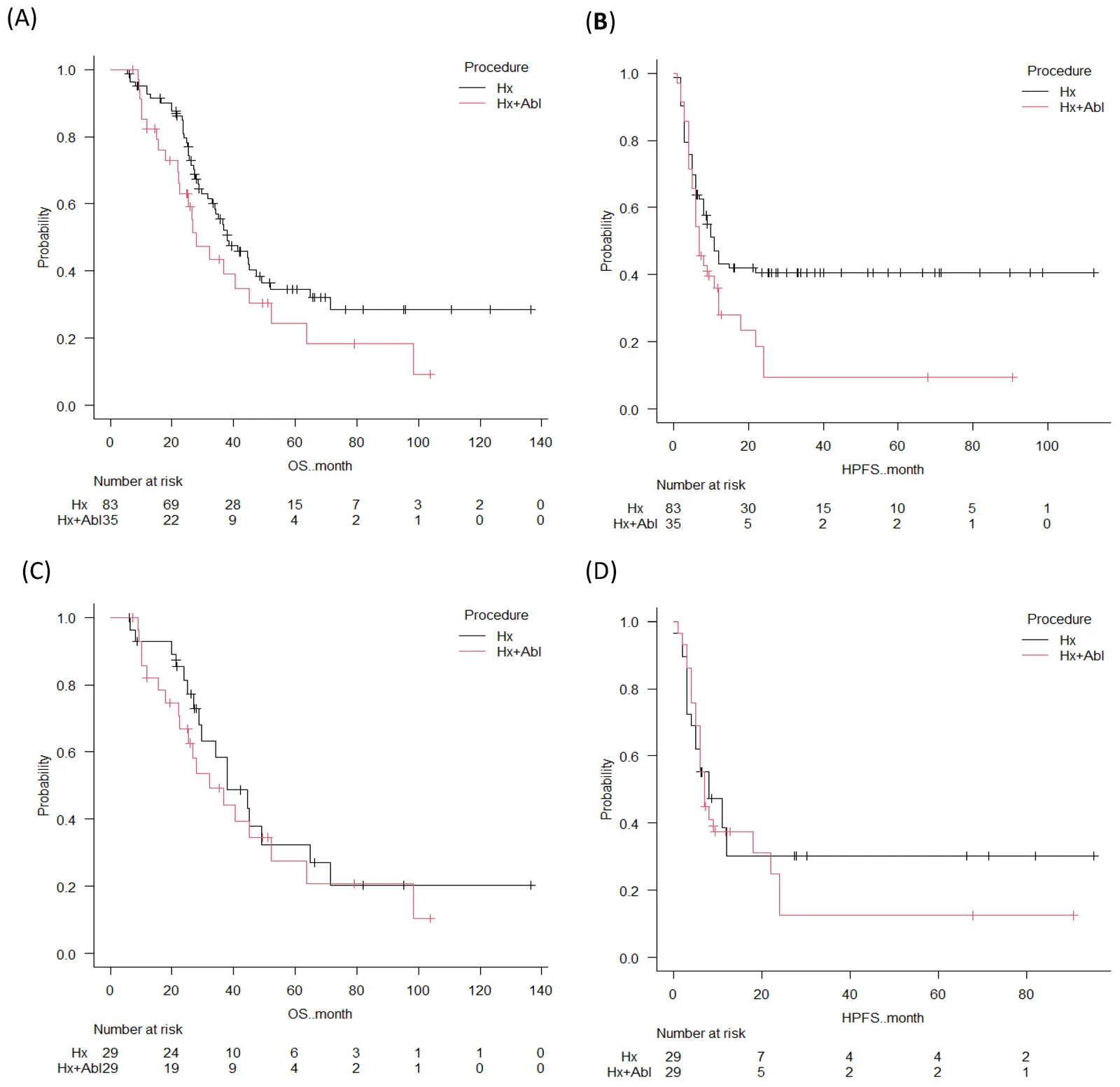
Overall survival and hepatic progression-free survival in patients undergoing hepatectomy alone or hepatectomy plus ablation, before and after propensity score matching. (**A**) Overall survival (OS) before propensity score matching. Patients who underwent hepatectomy alone (Hx, *n* = 83) showed a trend toward better OS than those who underwent hepatectomy plus ablation (Hx + Abl, *n* = 35); median 37.8 vs. 27.9 months (log-rank *p* = 0.097). (**B**) Hepatic progression-free survival (HPFS) before propensity score matching. HPFS was significantly shorter in the Hx + Abl group (*n* = 35) than in the Hx group (*n* = 83); median 11.0 vs. 7.0 months (log-rank *p* = 0.027). (**C**) Overall survival after propensity score matching. After matching (Hx *n* = 29; Hx + Abl *n* = 29), OS did not differ significantly between the two groups; median 44.5 vs. 36.8 months (log-rank *p* = 0.907). (**D**) Hepatic progression-free survival after propensity score matching. Post-matching HPFS showed no significant difference between the Hx and Hx + Abl groups; median 8.0 vs. 7.0 months (log-rank *p* = 0.581).

**Figure 3:**
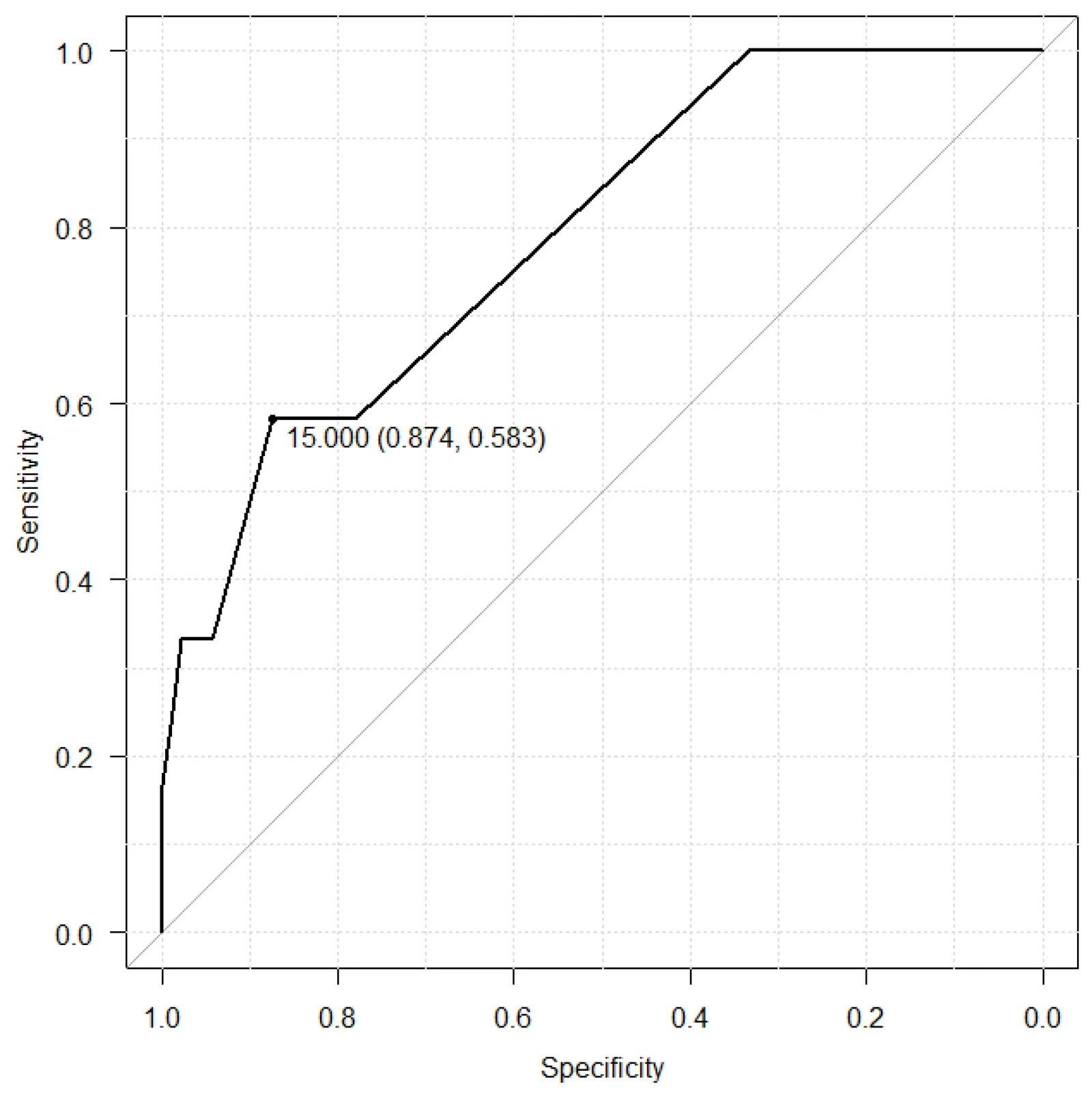
Receiver operating characteristic (ROC) curve of tumor size for predicting local recurrence. A total of 99 ablated lesions (MWA 96, RFA 3) were evaluated, with 12 local recurrences (12.2%). The optimal cutoff value was 15 mm, providing a sensitivity of 0.874 and specificity of 0.583. The area under the curve (AUC) was 0.791(95% CI: 0.665–0.916), indicating good discriminatory ability.

**Table 1:** Baseline characteristics before and after propensity score matching.

	Before PSM			After PSM		
Variables	Hx Group	Hx + Abl Group	*p*-Value	Hx Group	Hx + Abl Group	*p*-Value
	(*n* = 83)	(*n* = 35)		(*n* = 29)	(*n* = 29)	
Age (years)	67.0 ± 10.4	63.8 ± 10.9	0.137	62.3 ± 9.5	63.5 ± 10.9	0.672
Gender M vs. F	52(62.7)/31(37.3)	18 (51.4)/17(48.6)	0.307	18 (62.1)/11(37.9)	15 (51.7)/14(48.3)	0.596
Albumin (g/dL)	3.9 ± 0.4	3.8 ± 0.4	0.188	4.0 ± 0.4	3.8 ± 0.4	0.302
Total bilirubin (mg/dL)	0.7 ± 0.3	0.6 ± 0.3	0.275	0.6 ± 0.2	0.6 ± 0.3	0.923
AST (U/L)	24.6 ± 10.8	30.1 ± 13.4	0.019 *	21.5 ± 7.2	29.9 ± 13.6	0.005 *
Platelet count (×10^4^/μL)	20.0 ± 5.9	19.6 ± 9.5	0.785	19.7 ± 5.2	19.7 ± 10.1	0.997
PT (%)	96.0 ± 10.4	97.4 ± 12.0	0.544	97.2 ± 10.5	99.0 ± 12.2	0.551
ICG 15 min (%)	14.1 ± 11.7	14.3± 10.0	0.953	12.1 ± 7.0	14.4 ± 9.8	0.330
CEA (ng/mL)	58.6 ± 187.9	24.2 ± 35.7	0.286	18.2 ± 28.4	27.3 ± 38.6	0.311
CA19-9 U/mL	175.3 ± 538.1	493.1 ± 1964.2	0.177	183.0 ± 375.8	198.2 ± 686.8	0.917
APRI score	1.6 ± 1.0	1.9 ± 1.0	0.284	1.4 ± 0.5	1.8 ± 1.0	0.024 **
ALBI score	−2.6 ± 0.6	−2.6 ± 0.4	0.967	−2.7 ± 0.4	−2.6 ± 0.4	0.172
ALBI grade 1/2/3	43/40/0	18/16/1	0.408	19/10/0	2016/12/1	0.592
RAS wild vs. mutant	30 (56.6)/23(43.4)	17(54.8)/14(45.2)	1.000	14 (56.0)/11(44.0)	15(60.0)/10(40.0)	1.000
T (1/2/3/4)	2/3/57/21	0/3/21/11	0.477	0/1/23/5	0/3/17/9	0.267
N (0/1/2/3)	30/27/22/4	11/13/7/4	0.493	8/14/6/1	11/10/5/3	0.568
Main tumor size (cm)	3.6 ± 2.6	3.1 ± 1.7	0.289	3.1 ± 1.6	3.1 ± 1.8	0.998
Tumor number	4.5 ± 4.7	8.4 ± 6.8	<0.001 **	6.1 ± 5.5	6.5 ± 4.8	0.779
Synchronous vs. metachronous	53(63.9)/30(36.1)	25 (71.4)/10 (28.6)	0.525	21 (72.4)/8 (27.6)	21 (72.4)/8 (27.6)	1.000
Extrahepatic disease Y vs. N	22 (26.5)/61 (73.5)	9 (25.7)/26 (74.3)	1.000	9 (31.0)/20(69.0)	8 (27.6)/21 (72.4)	1.000
Preoperative chemotherapy Y vs. N	63 (75.9)/20 (24.1)	28 (80.0)/7(20.0)	0.811	25 (86.2)/4 (13.8)	22 (75.9)/7(24.1)	0.504
Postoperative chemotherapy Y vs. N	61(73.5)/22 (26.5)	32 (86.5)/5 (13.5)	0.230	25 (86.2)/4 (13.8)	24 (82.8)/5(17.2)	1.000
Beppu score	11.0 ± 4.1	13.5 ± 3.6	0.003 **	12.8 ± 4.6	12.9 ± 3.7	0.925
Beppu score Risk grade H/M/L	46/30/7	28/7/0	0.005 *	2018/9/2	19/10/0	0.623
ras-Beppu score Risk grade H/M/L	1952/9/4	31/4/0	0.401	2023/4/1	25/4/0	0.85
GAME score	3.0 ± 1.4	3.4 ± 1.3	0.064	3.2 ± 1.2	3.3 ± 1.3	0.918
GAME grade H/M/L	26/41/6	16/19/1	0.184	10/16/2	12/15/1	0.915
Tumor burden score	8.1 ± 5.7	11.5 ± 6.7	0.006 **	9.3 ± 5.5	9.7 ± 5.0	0.785
Hepatectomy ≥2 segment vs. others	51(61.4)/32(38.6)	22 (62.9)/13(37.1)	0.603	15 (51.7)/14 (48.3)	9 (31.0)/20 (69.0)	0.182
Operation time (min)	338.5 ± 111.2	351.4 ± 106.3	0.564	372.5 ± 111.6	344.1 ± 114.4	0.341
Blood loss (ml)	721.7 ± 776.2	1100.7 ± 1199.8	0.046 **	754.8 ± 641.0	1034.1 ± 1248.6	0.290
Hospital stay (day)	19.6 ± 12.5	24.4 ± 16.0	0.082	20.8 ± 13.2	24.5 ± 16.5	0.345
Resected liver weight (g)	335.1 ± 275.9	256.3 ± 237.9	0.185	466.9 ± 331.1	244.5 ± 250.9	0.015 **
Future liver remnant ratio (%)	72.1 ± 17.3	78.1 ± 13.5	0.070	64.2 ± 18.0	79.0 ± 13.1	0.001 **
Yamanaka’s prediction score	32.0 ± 17.6	25.3 ± 14.4	0.130	28.9 ± 12.0	26.7 ± 12.9	0.604
Major complication	19 (22.9)/	10 (28.6)/	0.376	8 (27.6)/	9 (31.0)/	1.000
CD ≥ 3 vs. CD ≤ 2	64 (77.1)	25 (71.4)		21(72.4)	20 (69.0)	
PHLF Grade A/B/C vs. none	36/4/1/47	13/0/0/24	0.640	12/3/1/13	11/0/0/18	0.174

PHLF: post-hepatectomy liver failure (International Study Group of Liver Surgery (ISGLS). * chi-square test, ** Student’s *t*-test.

**Table 2:** Univariate and multivariate analyses of overall survival.

Variables	*n*	Univariate Analysis	Multivariate Analysis		
		*p* Value	HR	95% CI	*p* Value
ALBI grade 1 vs. ≥2	61/57	0.405			
Synchronous vs. metachronous	78/40	0.155			
Extrahepatic lesion Y vs. N	31/87	0.165			
Tumor number ≥ 5 Y vs. N	36/82	0.051	1.45	0.877–2.400	0.148
RAS wild vs. mutant	47/37	0.192			
Lymph node metastasis Y vs. N	76/42	0.022	1.105	0.869–1.405	0.247
Preoperative chemotherapy Y vs. N	91/27	<0.001	3.92	1.746–8.801	<0.001
GAME risk grade High vs. others	41/77	0.031			
Beppu score risk grade High vs. others	66/52	<0.001			
ras-Beppu score risk grade High vs. others	83/31	0.052			
Treatment Hx vs. Hx + Abl	83/37	0.093			
PHLF Y vs. N	51/67	0.021			
Postoperative chemotherapy Y vs. N	91/27	0.275			
Intrahepatic recurrence Y vs. N	45/80	<0.001			

Preoperative variables with *p* < 0.10 in univariate analysis were entered into the multivariate Cox regression model to identify independent prognostic factors.

## Data Availability

The datasets generated and analyzed during the current study are available from the corresponding author upon reasonable request.

## Supplementary Materials

The following supporting information can be downloaded at: https://www.mdpi.com/article/10.3390/cancers18203386/s1, Figure S1: Subgroup analyses stratified by Beppu, ras-Beppu, and GAME scores with interaction. Panels (A–F) show Kaplan–Meier curves for overall survival (OS) and hepatic progression-free survival (HPFS) stratified by Beppu (A,B), ras-Beppu (C,D), and GAME (E,F) high- and low-risk categories in the propensity-matched cohort. Survival outcomes were compared between hepatectomy alone (Hx) and hepatectomy plus intraoperative ablation (Hx + Abl). Across all three scoring systems, no statistically significant differences in OS or HPFS were observed between Hx and Hx + Abl within either risk category (Beppu: OS *p* = 0.30, HPFS *p* = 0.70; ras-Beppu: OS *p* = 0.50, HPFS *p* = 0.90; GAME: OS *p* = 0.90, HPFS *p* = 0.60). Cox models incorporating interaction terms showed no evidence of heterogeneity of treatment effect (Beppu HR 1.43, *p* = 0.66; ras-Beppu HR 1.37, *p* = 0.69; GAME HR 2.29, *p* = 0.25).These findings indicate that hybrid hepatectomy performed similarly across prognostic strata, although subgroup analyses were limited by small sample sizes.
