# Supplementary material for: Outcomes of Combined Hepatectomy and Intraoperative Ablation for Multiple Colorectal Liver Metastases: A Single-Center Retrospective Study
Source: Cancers (Basel). 2026 Oct 20;18(20):3386. doi: 10.3390/cancers18203386 (PMC13702246; doi:10.3390/cancers18203386)
Supplement: Supplementary file 1 [file cancers-18-03386-s001.zip › cancers-4582230-supplementary.pdf]

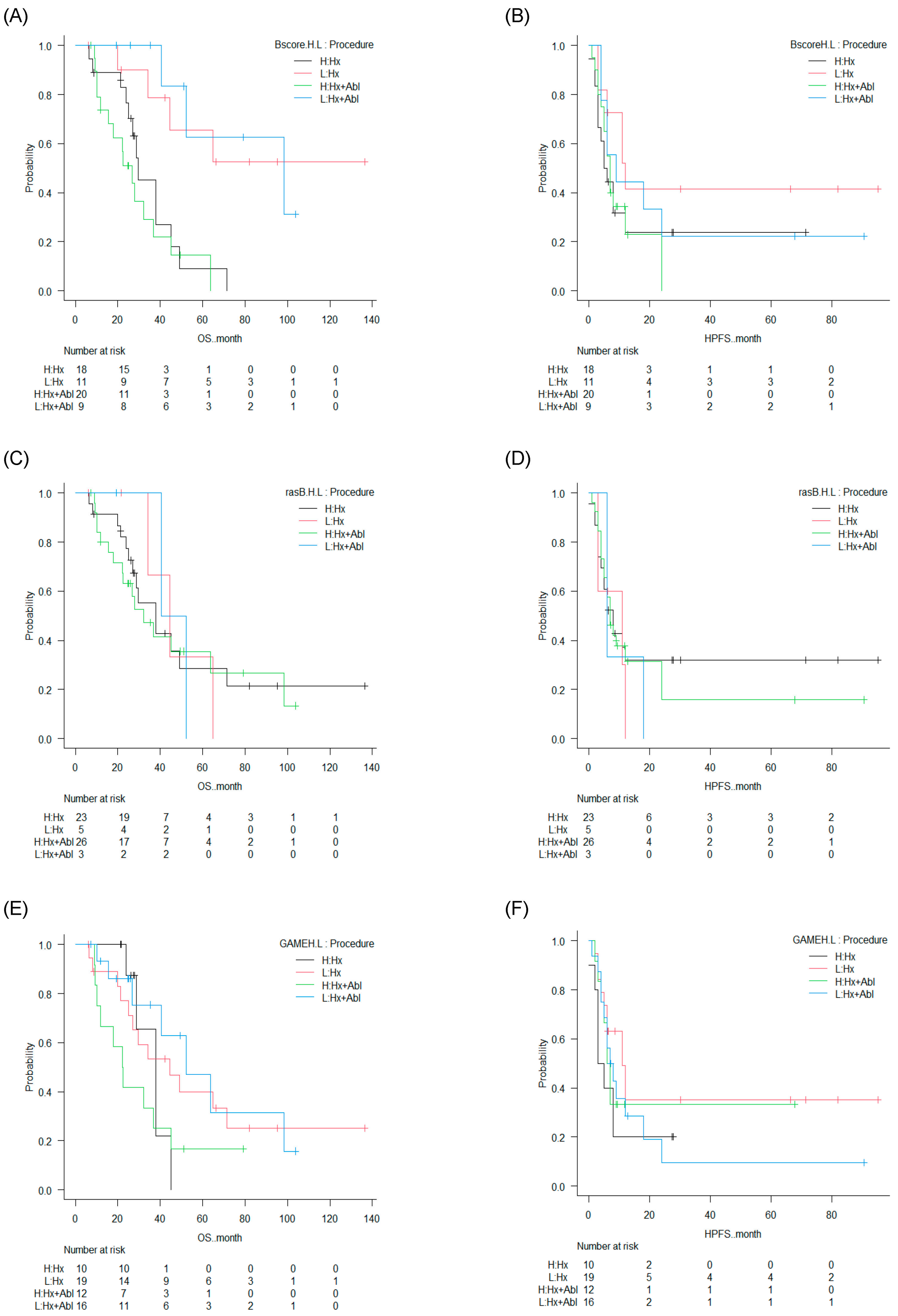

Supplementary Figure S1. Subgroup analyses stratified by Beppu, ras-Beppu, and GAME scores with interaction. Panels (A–F) show Kaplan–Meier curves for overall survival (OS) and hepatic progression-free survival (HPFS) stratified by Beppu (A,B), ras-Beppu (C,D), and GAME (E,F) high- and low-risk categories in the propensity-matched cohort. Survival outcomes were compared between hepatectomy alone (Hx) and hepatectomy plus intraoperative ablation (Hx + Abl). Across all three scoring systems, no statistically significant differences in OS or HPFS were observed between

Hx and Hx + Abl within either risk category (Beppu: OS $p = 0.30$, HPFS $p = 0.70$; ras-Beppu: OS $p = 0.50$, HPFS $p = 0.90$; GAME: OS $p = 0.90$, HPFS $p = 0.60$). Cox models incorporating interaction terms showed no evidence of heterogeneity of treatment effect (Beppu HR 1.43, $p = 0.66$; ras-Beppu HR 1.37, $p = 0.69$; GAME HR 2.29, $p = 0.25$).These findings indicate that hybrid hepatectomy performed similarly across prognostic strata, although subgroup analyses were limited by small sample sizes.
